# Anatomical variation in the branching pattern of the aortic arch: a literature review

**DOI:** 10.1007/s11845-022-03196-3

**Published:** 2022-10-22

**Authors:** Anna Murray, Eiman Abdel Meguid

**Affiliations:** 1 Queen’s University Belfast, University Road, Belfast, BT7 1NN UK; 2 School of Medicine, Dentistry and Biomedical Sciences, Whitla Medical Building, Queen’s University Belfast, 1st Floor, 97 Lisburn Road, Belfast, BT9 7AE UK

**Keywords:** Aberrant subclavian artery, Anatomical variation, Arch of aorta, Bovine arch, Brachiocephalic trunk, Vertebral artery

## Abstract

**Background:**

Many anatomical variations of the branching pattern of the aortic arch have been documented in the literature. These find their origin in alterations to the embryological development of the arch and have significant implications for surgical and radiological interventions.

**Methods:**

Embase and Medline database searches were carried out in June 2021 and identified 1197 articles, of which 24 were considered eligible.

**Results:**

Twenty-eight variations were found. The prevalence of the six main variations found is as follows: normal configuration (61.2–92.59%); bovine arch type 1 (4.95–31.2%); bovine arch type 2 (0.04–24%); origin of left vertebral artery (0.17–15.3%); aberrant right subclavian artery (0.08–3.33%); thyroid ima artery (0.08–2%). Concomitant variations present in conjunction with these variations are also documented, as were other variations which could not be classified into these six groups.

**Conclusions:**

Anatomical variations in the branching pattern of the aortic arch are present in over one-third of individuals in some populations. These are important pre- and intra-operatively during thoracic, neck and thyroid surgery. A greater effort should be employed to construct an official classification to facilitate greater understanding among clinicians.

## Introduction

**Fig. 1:**
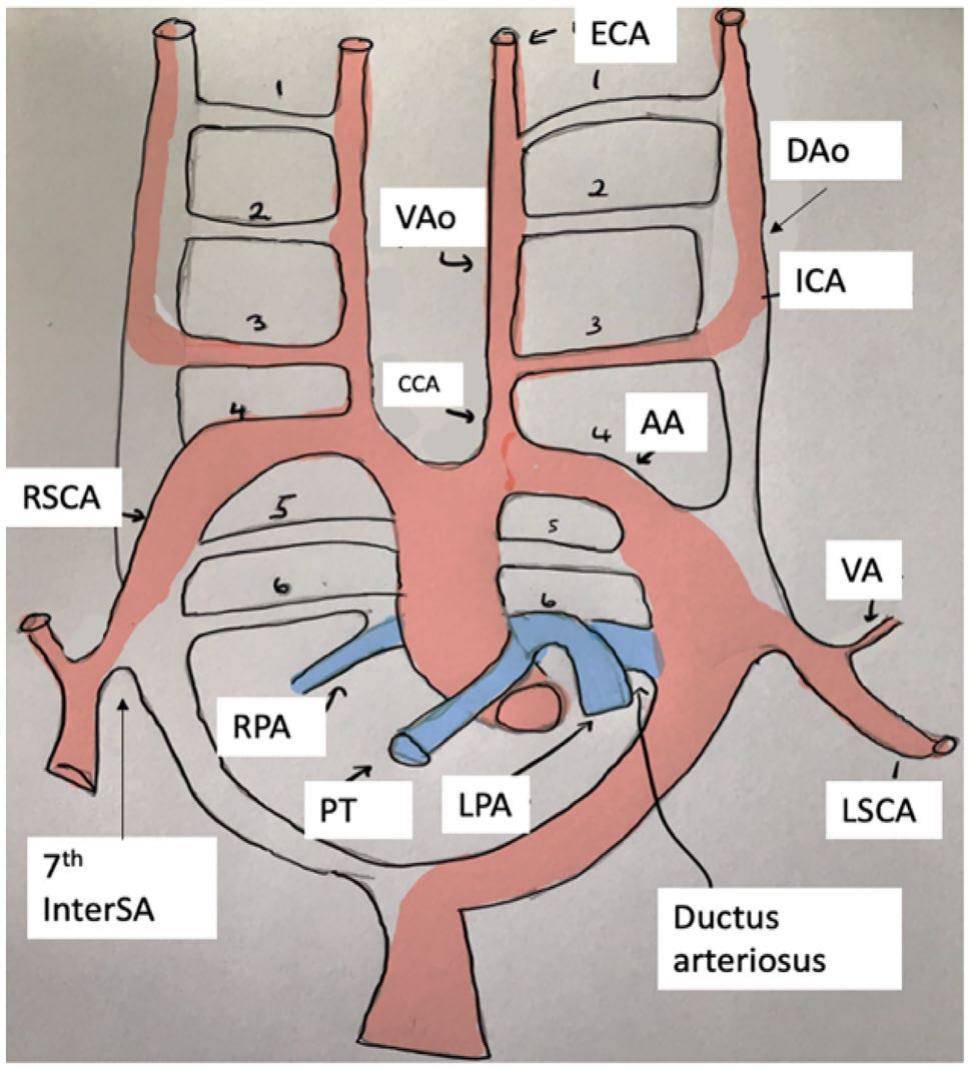
Self-drawn diagram outlining the development of the great vessels from the aortic arches. Numbers 1–6 represent the arches. RSCA, right subclavian artery; 7th InterSA, seventh intersegmental artery; RPA, right pulmonary artery; PT, pulmonary trunk; VAo, ventral aorta; CCA, common carotid artery; LPA, left pulmonary artery; ECA, external common artery; AA, aortic arch; DAo, dorsal aorta; ICA, internal carotid artery; VA, vertebral artery; LSCA, left subclavian artery. [5] Adapted from Jakanani and Adair

In the case of normal anatomy, the aortic arch (AA) gives rise to three main branches [1]. These are, from left to right, the brachiocephalic trunk (BCT), the left common carotid artery (LCCA) and the left subclavian artery (LSCA). The BCT further gives rise to the right common carotid (RCCA) and subclavian (RSCA) arteries. Each respective subclavian artery then gives rise to a vertebral artery (VA). Owing to the complex embryological development of the aortic arch (Fig. 1) and its branches, deviation from normal anatomy of the branching patterns is common and is usually asymptomatic, not detected until the patient undergoes imaging, surgery or autopsy [2, 3].

In 63.5–89.4% of individuals, the normal branching pattern described is present [4]. However, several variations of the branching pattern of the AA have been documented in the literature. These include the bovine aortic arch (the most common variant) [5] and origin of the left vertebral artery (LVA) from the AA (second most common variation) [4, 6]. In other instances, the RSCA arises as a branch of its own and not from the BCT, giving rise to a fourth branch from the arch [2]. In some individuals, the thyroid gland is supplied by a collateral branch, the thyroid ima artery, which arises directly as a branch from the aortic arch [7].

### Genetic correlation with anatomical variations

Anatomical variation of the branches of the AA has been linked to various chromosomal abnormalities. Up to 98.4% of paediatric patients with a bovine aortic arch have at least one congenital cardiac defect [8]. The aberrant right subclavian artery has been established as a biomarker for Down syndrome and other cardiac abnormalities, with a reported prevalence as high as 35% among individuals with Down’s syndrome [9].

### Anatomical variation of the branching patterns of the aortic arch

#### Variation involving the brachiocephalic trunk

The most common variation in aortic arch branching is known as the ‘bovine aortic arch’ (BA) [10]. There are two subtypes of this anatomical variant. Type 1 (BAT1), or the common ostium variant, is the most common type (up to 13%) and occurs when the LCCA and BCT originate from the same point [11]. The second (BAT2) (9%) occurs when the LCCA arises as a branch of the BCT itself [11]. The bovine AA has been reported to be present in 10–25% of individuals and comprises over two-thirds of all branching variations of the AA [12, 13].

#### Variant origin of vertebral arteries

The origin of one of the left vertebral arteries (LVA) from the AA has been reported to be present in 2.4–6.9% of the population, most commonly between the origin of the LCCA and LSCA (Fig. 5) [14]. Origin of the LVA from the aortic arch has also been associated with chromosome 22q11 deletion [15]. Rarely, there may be a duplication of the LVA arising from the AA [14]. In other instances, the LVA arises the last branch of the AA [6, 13].

#### Aberrant right subclavian artery (ARSA)

The ARSA, or ‘retro-oesophageal’ RSCA, arises as the last branch of the AA (Fig. 6) [16]. In the case of a present ARSCA, the RSCA is absent from the BCT. An ARSCA, also known as arteria lusoria, may have clinical implications owing to its relations to the oesophagus, resulting in possible oesophageal compression [16]. Presence of an ARSCA may be associated with diverticulum of Kommerell in up to 60% of cases [17].

#### Thyroid ima artery

The thyroid ima artery is an accessory artery which may be present as a collateral supply to the thyroid gland. If present, it arises from the AA or BCT (Fig. 6) [7]. This variation may be present in up to 15% of individuals [18]. The vessel may also originate from the RCCA, the internal mammary, subclavian or inferior thyroid arteries [13].

#### Rare variations in branching of the aortic arch

Variations not previously discussed occur very rarely (< 0.5%) [13]. Variation can result in two-, three-, four- or five-branched variants. These will be discussed throughout this writing.

### Objective

The purpose of this project was to review the literature relating to cadaveric, radiological and surgical studies of the anatomical variations in the branching patterns of the aortic arch. Furthermore, it investigated the clinical implications of these anatomical variations regarding the planning and execution of surgical and radiological procedures involving the arch and its branches.

## Materials and methods

Searches were carried out in June 2021 on EMBASE and MEDLINE using the following search terms: Anatomical variation/Anatomic variation and Arch of aorta/Aortic arch; Anatomical variation/anatomic variation and Brachiocephalic trunk/Brachiocephalic artery/innominate artery/Bovine; Anatomical Variation/Anatomic Variation and Vertebral artery and Arch of the Aortic/Aortic Arch and Origin; Anatomical variation/Anatomic variation/Aberrant and Origin and Subclavian Artery and Arch of the Aorta/Aortic Arch; Thyroid ima artery and Origin and Aortic Arch/Arch of the Aorta; Anatomical variation/Anatomic Variation/malformation and Aortic Arch/Arch of the aorta and Brachiocephalic trunk/Brachiocephalic artery/innominate artery/Bovine or Vertebral Artery or Subclavian Artery or Common Carotid Artery or Thyroid ima; Anatomical variation/Anatomic Variation/malformation and Aortic Arch/Arch of the aorta and Brachiocephalic trunk/Brachiocephalic artery/innominate artery/Bovine or Vertebral Artery or Subclavian Artery or Common Carotid Artery or Thyroid ima and Branching.

### Inclusion criteria

Studies included were those which were:

- originally published in English
- published between the year 2000 and the current year
- whose subject of interest was relevant to anatomical variation of the branching pattern in the aortic arch including cadaveric, radiological and surgical papers
- with a sample size greater than 50

### Exclusion criteria

Those studies excluded were:

- published in another language or prior to the year 2000
- case reports or specimens with evident pathology involving the aortic arch and/or its branches or who had diagnosed congenital heart defects
- those that examined foetal variations of the AA
- review articles, letters, notes to the editor or abstract-only conference abstracts
- studies whose focus was vascular ring or variation of the arch itself, e.g. a right AA, double AA or Kommerell’s diverticulum

### Selection of studies

**Fig. 2:**
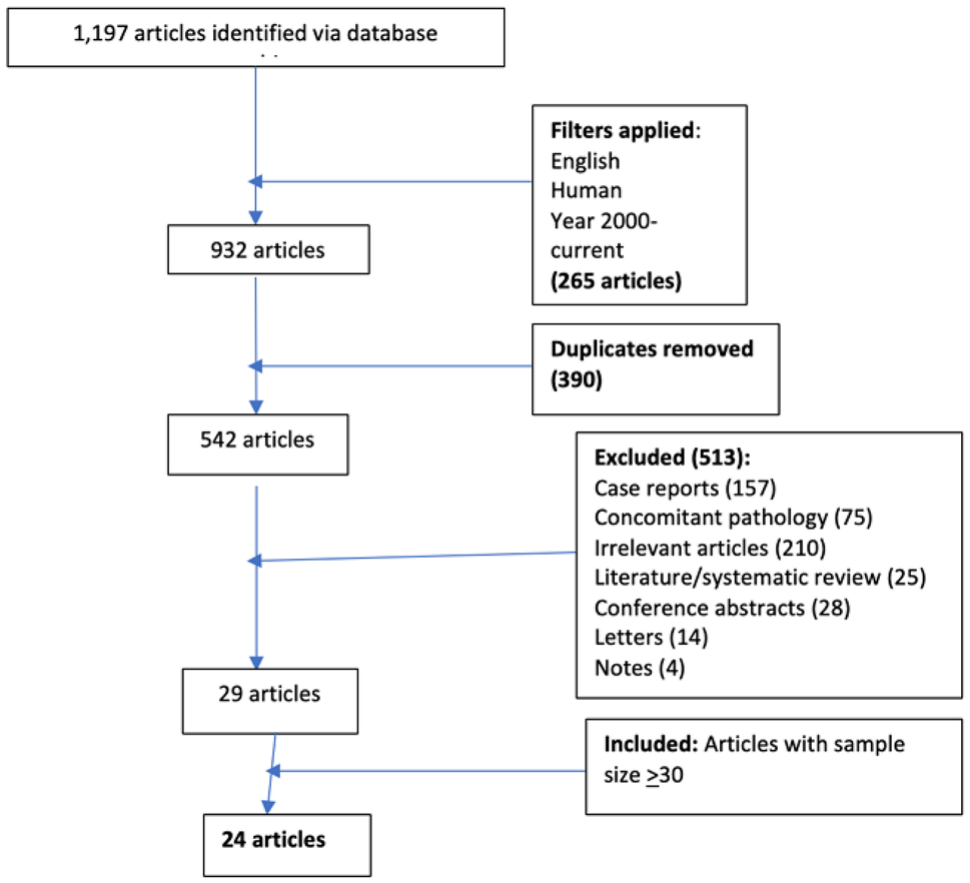
Flowchart illustrating selection of studies

Searches identified 1197 articles which were reviewed for eligibility. Eight hundred twenty-one were identified by EMBASE and 376 were identified by MEDLINE. After applying inclusion and exclusion criteria, twenty-four articles were selected (Fig. 2, Table 1).

**Table 1:** Studies examined in this review including sample characteristics

**Study**	**Modality**	**Sample**	**Age**	**Male**	**%**	**Female**	**%**	**Focus**	**Ethnicity**
**Aboulhoda et al.** [19]	CT angiogram	100						All	Egyptian
**Acar et al.** [20]	CT angiogram	96	31–86	64	66.67	32	33.33	All	
**Bhatia et al.** [21]	Cadaveric	81		18	22.2	63		All	South Australian
**Budhiraja et al.** [22]	Cadaveric	52						All	Central Indian
**Ergun et al.** [23]	CT angiogram	1001	18–88	519	51.8	482	48.2	All	
**Gupta and Sodhi** [24]	Cadaveric	100	40–70					All	
**Indumathi et al.** [25]	CT angiogram, dissection and autopsy	73		46	63	27	37	All	South Indian
**Jakanani and Adair** [5]	CT angiogram	861						All	
**Karacan et al.** [37]	CT angiogram	1000	17–94	610	61	390	39		Turkish
**Keet et al.** [27]	Cadaveric	733	20–102	516	70.4	217	29.6	All	African
**Kondori et al.** [28]	MR angiogram	266						All	
**Meyer et al.** [3]	CT angiogram	178	< 1					BA	
**Mustafa et al.** [33]	CT angiogram	500	2–92	291	58.2	209	41.8	All	Jordanian
**Natsis et al.** [36]	Cadaveric	267	53 ± 13	126	47.19	141	52.81	ARSCA	Greek
**Omotoso et al.** [38]	CT angiogram	554	10–99	307	55.4	247	44.6	LVA	South African
**Piyavisetpat et al.** [34]	CT angiogram	687	18–94	361	52.55	326	47.45	All	Thai
**Qiu et al.** [35]	Cadaveric	120	5–85	91	75.83	29	24.17	All	Chinese
**Syperek et al.** [29]	CT angiogram	322	29–94	172	53.42	150	46.58	BA	
**Tapia-Nanez et al.** [30]	CT angiogram	220	≤ 18	114	51.82	106	48.18	All	Mexican
**Tardieu et al.** [39]	Cadaveric	50		28	56	22	44	LVA	
**Vinnakota and Bhattam** [31]	CT angiogram, dissection and autopsy	435						All	Southeast Indian
**Wang et al.** [32]	CT	2370	18–88	1348	56.9	1022	43.1	All	Chinese
**Woraputtaporn et al.** [14]	Cadaveric	266						LVA	Thai
**Yamaki et al.** [40]	Cadaveric	511		303	59.3	212	41.5	LVA	Japanese

## Results

Twenty-eight anatomical variations were found, including 10,843 specimens; 56.36% of specimens (*N* = 4977) were male and 41.42% female (*N* = 3675). Absent data is accounted for as some studies did not specify sex of specimens under investigation.

**Fig. 3:**
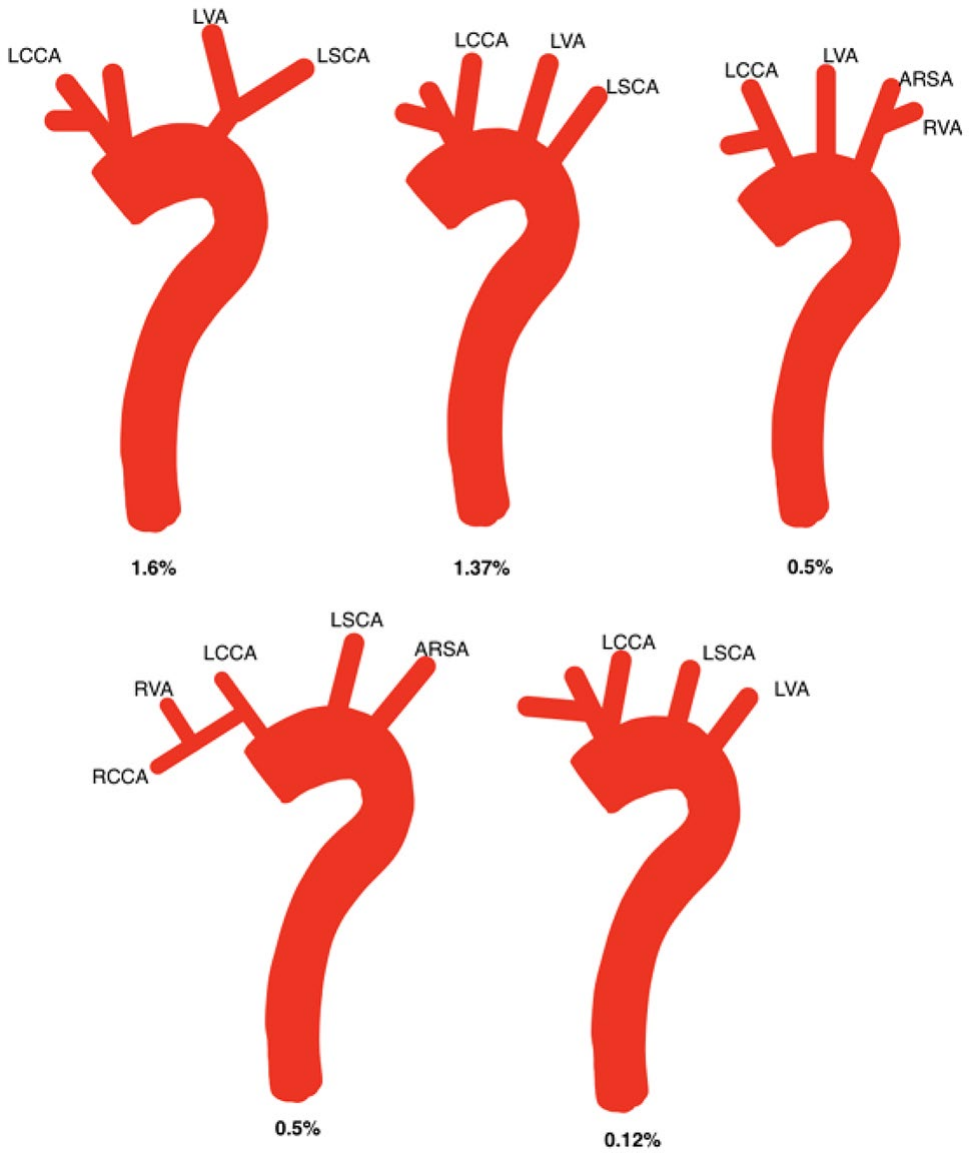
Self-drawn images illustrating additional branching patterns exhibited alongside the bovine arch variation. BAT1, bovine arch type 1; LVA, left vertebral artery; LSCA, left subclavian artery; RCCA, right common carotid artery; LCCA, left common carotid artery; ARSA, aberrant right subclavian artery

**Fig. 4:**
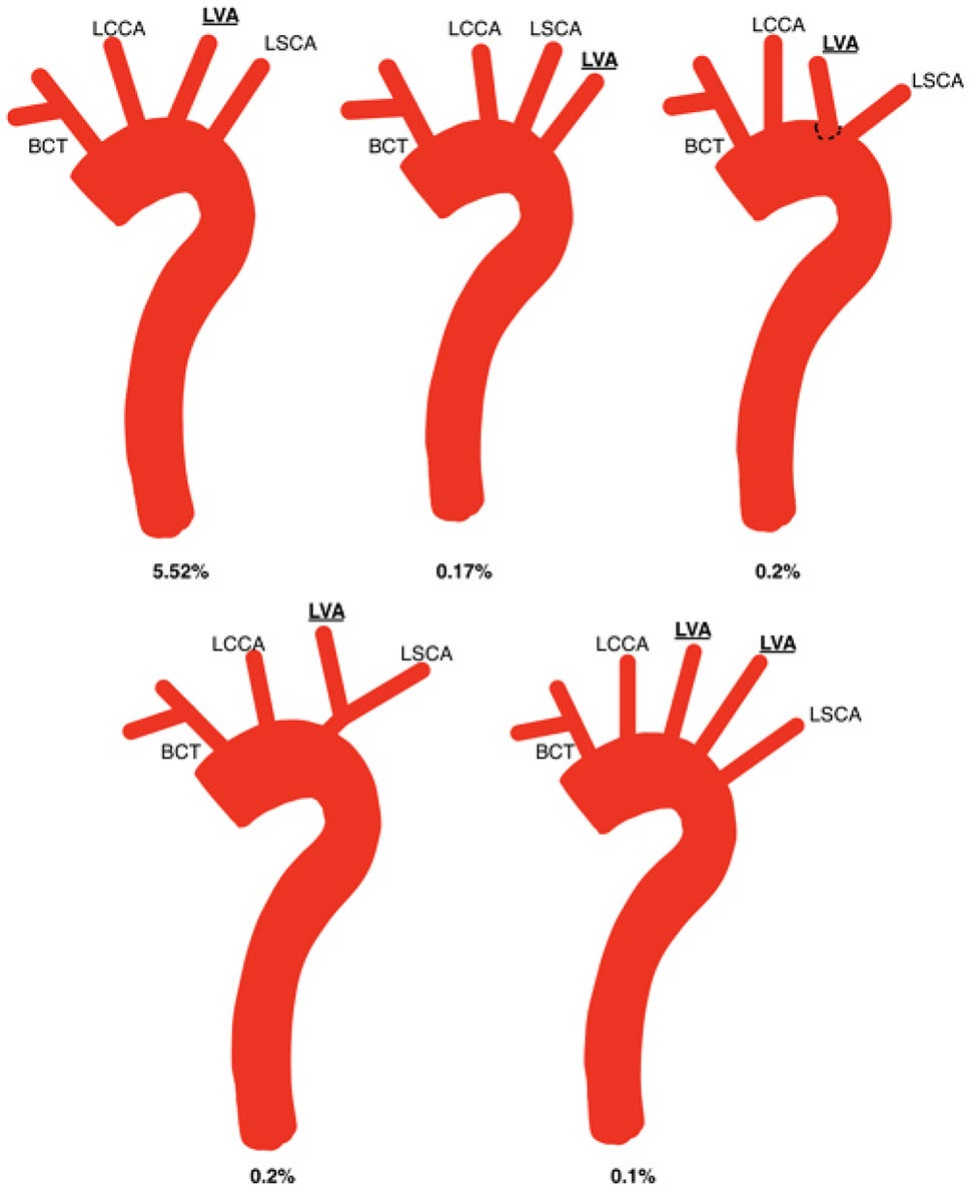
Self-drawn images to represent average percentage prevalence of the origin of the left vertebral artery (LVA) from the AA. LVA has been highlighted in yellow. From top left to bottom-right: BCT, LVA, LCCA, LSCA; BCT, LCCA, LSCA, LVA; BCT, LCCA, LVA dorsal to LSCA; BCT, LCCA, common trunk for LVA and LSCA; BCT, LCCA, duplication of LVA, LSCA

**Fig. 5:**
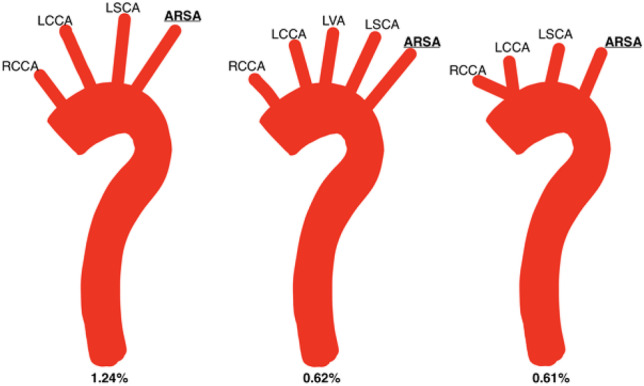
Self-drawn images to represent average percentage prevalence of anatomical variation of the AA involving the aberrant right subclavian artery (ARSA). The ARSCA has been highlighted in yellow. From left to right: RCCA, LCCA, LSCA, ARSA; common origin of carotids (arrow), LSCA, ARSA; RCCA, LCCA, LVA, LSCA, ARSA

**Fig. 6:**
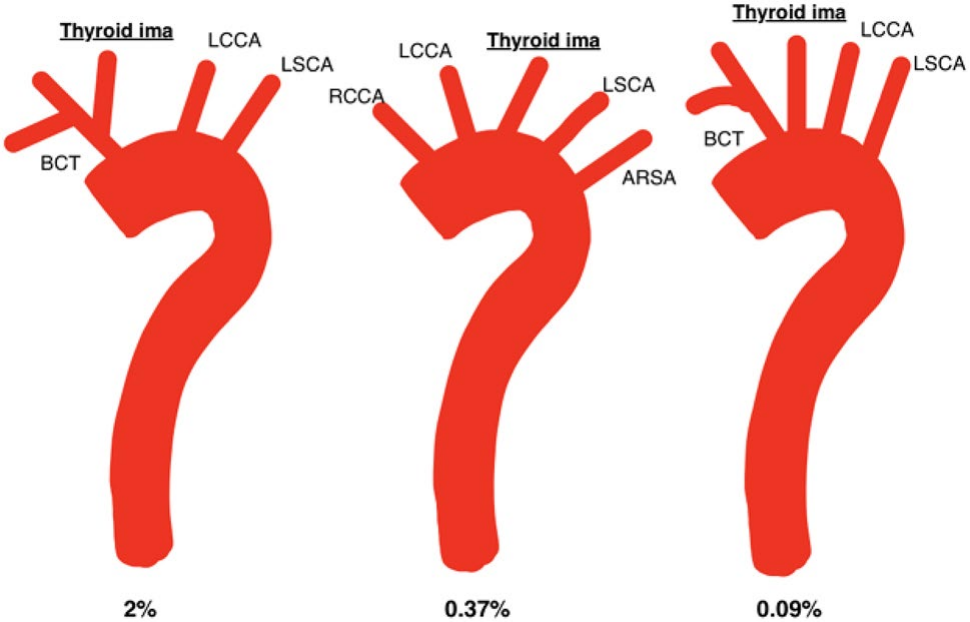
Self-drawn images to illustrate anatomical variations of the branching patterns of the aortic arch with the regard to the thyroid ima artery (highlighted and underlined). BCT, brachiocephalic trunk; LCCA, left common carotid artery; LSCA, left subclavian artery; RCCA, right common carotid artery; ARSA, aberrant right subclavian artery

Five main branching variations were found among the studies. Table 2 outlines these and their range of prevalence. They included the normal arrangement of the branches (Table 3), BA (Table 4 and Fig. 3), LVA originating from the AA (Fig. 4), ARSCA (Fig. 5) and thyroid ima arising from AA (Fig. 6).

**Table 2:** Range of the five main variations found in this review and their associated studies

**Variation**	**Lowest**	**Highest**
**Normal**	61.2% [33]	92.59% [21]
**Bovine type 1**	4.95% [38]	31.2% [33]
**Bovine type 2**	0.04% [32]	24% [19]
**Left vertebral artery arising from AA**	0.10% [23]	15.3% [22]
**Aberrant right subclavian artery**	0.08% [32]	3.33% [35]
**Thyroid ima artery**	0.08% [32]	2.0% [24]

**Table 3:** Prevalence of the normal pattern of branching

**Normal branching pattern**	**Total**	**Male**	**Female**
**Total number of specimens**	7187	2360	1437
**Average percentage**	78.76	73.06	59.14

**Table 4:** Prevalence of bovine arch subtypes including relevant studies

**Variation**	**Reported in study**	**Total prevalence**	**Male prevalence**	**Female prevalence**
**Bovine arch type unspecified**	Meyer et al. [3] Syperek et al. [29]	143 (21.06%)	32 (18.6%)	23 (15.3%)
**Bovine arch type 1**	Aboulhoda et al. [19] Acar et al. [20] Budhiraja et al. [22] Gupta and Sodhi [24] Indumathi et al. [25] Keet et al. [27] Kondori et al. [28] Mustafa et al. [33] Piyavisetpat et al. [34] Wang et al. [32]	882 (12.90%)	233 (11.12%)	151 (12.02%)
**Bovine arch type 2**	Aboulhoda et al. [19] Ergun et al. [23] Karacan et al. [37] Piyavisetpat et al. [34] Syperek et al. [29] Wang et al. [32]	267 (8.66%)	110 (14.08%)	62 (9.51%)

### Normal branching pattern of the aortic arch

Eighteen studies reported cases exhibiting the normal configuration of the branching pattern of the AA [5, 19–35]. A total of 7187 specimens were reported to have this normal configuration of branching (average percentage prevalence: 78.76%). Of these 7187 specimens, 2360 were male (average male percentage prevalence: 73.06%) and 1437 were female (average female percentage prevalence: 59.14%) (Table 3).

### Aberrant right subclavian artery

Forty-one specimens (average prevalence: 1.24%) had the configuration RCCA, LCCA, LSCA and ARSA out of the eleven studies which reported this variation [20, 23, 25, 26, 31–35]. In these eleven studies, equal numbers of males [7] and females [7] were reported (average male prevalence 1.53% vs 1.41% average percentage female prevalence).

Ten specimens across two studies exhibited the variation RCCA, LCCA, LVA, LSCA and ARSA (average percentage prevalence: 0.62%) [32, 34]. Of the specimens whose sex was reported in these two studies, one male (average percentage prevalence: 0.28%) and seven females (2.15%) demonstrated this variation.

Ten studies also reported the variation in which there was a common origin of the common carotid arteries, then the LSCA, then the ARSA as the last branch to arise from the AA [20, 23, 26, 30–36].

Across the studies, twenty-six specimens exhibited these variations (average prevalence: 0.61%). Of the studies in which sex of specimens was reported, seven males and nine females displayed this variation (average male percentage prevalence: 2.73% vs 1.45% female). These configurations, alongside their respective average prevalence, are shown in Fig. 5.

### Thyroid ima artery

Three cases of the thyroid ima artery arising directly from the AA were reported across two studies [26, 32]. The average prevalence of this variation was 0.09% and was reported in one male by Karacan et al. [26]. Gupta and Sodhi reported two cases of the thyroid ima arising from the BCT [24]. Additionally, Natsis et al. [36] reported one male (average male prevalence: 0.71%) who exhibited the thyroid ima artery arising from the AA with a concomitant ARSA (average prevalence: 0.37%).

### Other variations

Eleven other rarer variations were also found which could not be categorised into the six main categories discussed in Table 2. These, the total number of specimens exhibiting the variation (*N*) and their average prevalence, are listed below:

1. BCT and common trunk for LCCA and LSCA (*N* = 4; average prevalence: 3.33%) [24, 27, 33].
2. AA gives off a mediastinal branch (*N* = 2; average prevalence: 2%) [24].
3. Common trunk for BCT and left internal carotid artery, LCC, LSCA (*N* = 1; average prevalence: 1.06%) [20].
4. RSCA, RCCA, LCCA, LSCA (*N* = 10; average prevalence: 0.70%) [23, 31].
5. Origin of RVA from RCCA (*N* = 6; average percentage prevalence: 0.61%) [31, 34].
6. Trifurcation of BCT into RCCA, RVA and RSCA; LCCA; LVA; LSCA (*N* = 2; average percentage prevalence: 0.29%) [34].
7. Bicarotid trunk (*N* = 1; average percentage prevalence: 0.23%) [31].
8. BCT gives off mediastinal branch (*N* = 2; average percentage prevalence: *N* = 1; average percentage prevalence: 2%) [24].
9. RCCA, LICA, LECA, LSCA, RSCA (*N* = 1; average percentage prevalence: 1.06%) [20].
10. RSCA, RCCA, BCT (*N* = 1; average percentage prevalence: 0.1%) [23].
11. RVA originates from RCCA; ARSA present (*N* = 1; average percentage prevalence: 0.1%) [23].

Prevalence of these variations among the relevant studies, alongside their prevalence in male versus female specimens (if specified), is listed in Table 5. In the case of variation [1], two branches arose from the AA. In the case of [2], [3], [5], [8] and [10], three branches arose from the AA. In the case of [4], [6], [7] and [11], four branches arose from the AA. In the case of variation [9], five branches arose from the AA.

**Table 5:** Rarer variations found within this review and the associated studies which reported these variations

**Variation**	**Studies**	**Total Number of Specimens in Study**	**Male Prevalence**	**Female Prevalence**
**BCT and common trunk for LCCA and LSCA**	Gupta and Sodhi [24]	2 (2%)		
	Keet et al. [27]	17 (7.8%)		
	Mustafa et al. [33]	1 (0.2%)	1 (0.34%)	
**Common trunk for BCT and LICA, LCCA, LSCA**	Acar et al. [20]	1 (1.06%)		
**RSCA, RCCA, LCCA, LSCA**	Ergun et al. [23]	7 (0.7%)		
	Vinnakota and Bhattam [31]	3 (0.69%)		
**Origin of RVA from RCCA**	Piyavisetpat et al. [34]	2 (0.29%)	1 (0.28%)	1 (0.31%)
	Vinnakota and Bhattam [31]	4 (0.92%)		
**Trifurcation of BCT into RCCA, RVA and RSCA, LCCA, LVA, LSCA**	Piyavisetpat et al. [34]	2 (0.29%)	1 (0.28%)	1 (0.31%)
**Bicarotid trunk**	Vinnakota and Bhattam [31]	1 (0.23%)		
**Mediastinal branch from BCT**	Gupta and Sodhi [24]	2 (2%)		
**AA gives off mediastinal branch**	Gupta and Sodhi [24]	2 (2%)		
**RCCA, LICA, LECA, LSCA, RSCA**	Acar et al. [20]	1 (1.06%)		
**RSCA, RCCA, BCT**	Ergun et al. [23]	1 (0.1%)		
**RVA originates from RCCA; ARSA present**	Ergun et al. [23]	1 (0.1%)		

## Discussion

### Normal branching pattern

The normal branching pattern of the AA was the most common configuration across all studies (average: 78.76%) (Table 3). Percentage prevalence ranged from 61.2% [33] to 95.17% [31].

Studies in which sex was specified, with exception of the study by Aboulhoda et al. [19], found a male predominance (73.06% vs 59.14%—males vs females, respectively). This would inversely suggest that variation in the branching pattern of the AA is more common in females. Prevalence of the normal pattern of branching is most likely under-reported as studies with a specific area of focus neglected to report other variations. For example, Natsis et al. [36] only commented on the six cases which exhibited an ARSA out of 267 specimens included in the study and did not report any other variation or normal branching pattern. Likewise, Omotoso et al. [38], Tardieu et al. [39] and Yamaki et al. [40]—whose focus of study was the origin of the LVA from the AA—only reported findings of variation and not of normal anatomy.

There may be racial differences between patterns. Prevalence of the normal configuration was as high as 92.59% in the South Australian population studied by Bhatia et al. [21]. This contrasts with 65.2% in the African population studied by Keet et al. [27] and 61.2% in the Jordanian population studied by Mustafa et al. [33]. However, the study by Bhatia et al. was underpowered (*N* = 81) and all cadavers were of European descent [21]. This same study correlated rates of BA to socio-economic factors, suggesting that these may play a role in determining prevalence of the bovine arch in a population.

### Bovine aortic arch

Presence of a BA was the second most common arrangement (BAT1 range: 4.95–31.2%; BAT2 range: 0.04–24%). Specimens in this review exhibited a range of concomitant variations alongside the BA (Fig. 3). The most common variation was the common origin of the trunk and the LCCA (BAT1) (average prevalence: 12.90%), followed by the LCCA originating as a branch of the BCT (BAT2) (average prevalence: 8.66%). This is consistent with the literature [11].

Several papers failed to categorise the type of BA; however, in these cases, type was determined by description of the variation. Studies by Meyer et al., Tapia-Nanez et al. and Syperek et al. failed to specify type of BA or elaborate on the precise origin of the LCCA, limiting this review [3, 29, 30]. Data from these studies has been recorded as ‘BA unspecified’ in Table 4.

There are some queries regarding additional variations of the BA (Fig. 3). Variations recorded in Jakanani and Adair mention BA with concomitant ARSA [5]. However, this suggests that the BCT is not a true BCT as the RSCA originates as a separate branch and not within the trunk. Perhaps this variation would be better classified as ‘Bi-carotid trunk plus ARSA’.

With regard to differences in BA incidence among sexes, this review found that there was no clear overall pattern. Incidence of BAT1 exhibited a slight female predominance (12.02 vs 11.12% in females versus (vs) males, respectively). BAT2, however, was more prevalent in males (14.08% versus 9.51% in males vs females, respectively). With regard to studies in which the BA type was unspecified, Syperek et al. was the only study to specify incidence with regard to sex [29]. Taking data from this single study into account, males had a greater incidence of unspecified type of BA vs females (18.6% vs 15.3%, males vs females, respectively). Karacan et al. [37] found no difference in incidence of BAT2 between sexes (14.1%). In contradiction, Indumathi et al. [25] and Mustafa et al. [33] found BAT2 to be more prevalent in males. Based on these results, it is difficult to comment on sexual differences in prevalence of this variation and their subtypes. Perhaps a greater number of studies, particularly those specifying incidence among the respective sexes, would help distinguish difference in incidence.

### Origin of the left vertebral artery from the aortic arch

The LVA arose from the AA in five different locations, with a combined average prevalence of 6.19% (Fig. 4). Variation of the LVA was also seen in conjunction with the BA (Fig. 3). The most common arrangement was BCT, LCCA, LVA and LSCA (average: 5.52%) which was more prevalent in males than in females (7.60% vs 3.69%). This was the most common arrangement found by Qiu et al. (7.5%) who studied Chinese cadavers [32, 35]. This high prevalence may be due to cadaver ethnicity or small sample size (100) as a Chinese study by Wang et al. found the BAT1 to be the most common variation [32]. Neither study commented on genetic linkage between specimens which may have affected rates of prevalence.

The study of this variation was limited by lack of studies relating prevalence to sex (9/22 studies). While examining studies that did specify sex, it was found that there was a male predominance of this variation (7.6% vs 3.69%, male and female, respectively).

Two rarer variations were reported only by Mustafa et al. (33), with only one case each being reported (0.2%) (Fig. 4) [33]. In one case, the LVA arose dorsal to the LSCA. In the other case, there was a common trunk for the LVA and LSCA. This may suggest that these variations are exclusive to the Jordanian population. Interestingly, Ergun et al. reported one case (0.1%) of duplication of the LVA, which is considered a rare variation [23].

Small sample sizes may have affected the rates of LVA arising from the AA in this review. In the study by Bhatia et al. (*N* = 81), the origin of the LVA from the AA was the only variation reported (7.41%) [21]. The prevalence of this variation was 15.3% in the study by Budhiraja et al., which only examined 52 specimens [22].

### Aberrant right subclavian artery

Presence of the ARSA was more common in males than females in the absence of other variation (1.42% vs 0.67%, respectively), except when present alongside LVA arising from the AA (0.28% vs 2.15%, males and females respectively). This is unusual seeing that this review found the origin of the LVA from the AA as an isolated variation to be more prevalent in males. Within the literature, five arrangements involving the ARSA were reported (Fig. 5 and Table 5).

### Other types of variations

The most common variation found which cannot be categorised into the previous groups was a two-branched variant, where there was a BCT and a common trunk giving rise to the LCCA and LSCA (average prevalence: 3.33%). This variation was found in studies by Keet et al. [27] and Mustafa et al. [33], who studied African and Jordanian populations, respectively, suggesting a geographical correlation.

Other rarer variations were reported by studies contained in this review (Table 2). Gupta and Sodhi [24] reported two cases (2%) where the AA gave off a mediastinal branch. The apparent high prevalence of this variation may be due to the small sample size of this study (*N* = 100).

### Clinical importance of anatomical variations of the branching pattern of the aortic arch

In most cases, presence of variation does not result in loss or alteration of function and therefore does not require intervention [35].

#### Bovine arch

Ligation of an undetected BA may result in major ischaemic complications due to absence of supply to RSCA, RCCA, LCCA and RVA. BA has also been associated with concomitant abnormalities of the heart, AA aneurysms and aortic dissection [41, 42]. Presence of a BA may influence surgical approach. Meyer et al. hypothesised higher rates of coarctation in those with BA due to shorter clamping distances caused by the BCT being displaced distally and the LCCA moving more proximally [3]. Additionally, a BA makes transfemoral stenting of the LCCA more difficult due to the two tight turns from AA to the BCT and then into the LCCA. This has led to an approach from the upper limb being preferred [43].

#### Aberrant right subclavian artery

Due to its retro-oesophageal (83%) or tracheo-oesophageal course (16.7%), an ARSA may result in symptoms of dyspnoea or dysphagia [36]. Anatomical relations of the ARSA to the inferior laryngeal nerve or trachea necessitate caution during tracheostomy or thyroid surgery [44]. Additionally, the ARSA has an association with the diverticulum of Kommerell, a bulge in the arch itself [36].

#### Left vertebral artery from the aortic arch

This variation has been associated with a higher level of entry of the LVA into the transverse foramina of the cervical spine [14]. This has implications for cervical, spinal and vascular surgery, leaving the VA more exposed for a greater length [40]. If pre-operative imaging of the VA is neglected, severe neurological sequelae may result [14].

It has been found that, in cases with a BA alongside the LVA arising from AA, the BCT deviated to the left of the midline to compensate and therefore may be injured in anaesthetic procedures, e.g. tracheostomy [22, 45].

There has also been an association between posterior circulation ischaemia and origin of the LVA from the AA [46]. Wang et al. found that, in patients in whom the LVA originated from the AA, there was hypoplasia of the artery, diminishing blood flow in the vessel and therefore reducing posterior circulation [47]. Tulshidas Patil et al. [48] found death secondary to cerebrovascular disease to be more common among cadavers with variation in the LVA origin (normal variation vs variation in LVA origin: 12% vs 23.5%).

## Conclusion

Anatomical variation in the branching pattern of the arch of the aorta is not uncommon but is usually asymptomatic; not being detected unless the individual undergoes radiological investigation, surgery or autopsy. In several of the studies included, anatomical variation of the branching pattern comprised one-third of specimens, illustrating its high prevalence. This review found five main branching patterns reported among the literature. These included the bovine arch, the origin of the left vertebral artery from the aortic arch, an aberrant right subclavian artery and the thyroid ima artery arising from the arch.

The presence of a variation of the aortic arch is important clinically—not only from its use as an antenatal biomarker but also to its significance during thoracic, neck and thyroid surgery. It is essential that the clinician is aware of the possible presence of variant anatomy to prevent ischaemia of important structures or organs or to attribute a diagnosis to various symptoms such as dysphagia or dyspnoea.

Having carried out research in this field, the author recommends greater effort be employed to construct and consolidate an official classification system for the various anatomical variations of the branching pattern of the aortic arch. Further research in this area would facilitate greater understanding of the variations and better communication between multi-disciplinary teams and departments such as radiology and various surgical specialities for safer operative procedures.

## Data Availability

All data was obtained from studies 32–39 included on the reference list.

## Abbreviations

AA: Aortic arch
ARSA: Aberrant right subclavian artery
BA: Bovine arch
BAT1: Bovine arch type 1
BAT2: Bovine arch type 2
BCT: Brachiocephalic trunk
LCCA: Left common carotid artery
LSCA: Left subclavian artery
RSCA: Right subclavian artery
RCCA: Right common carotid artery
